# Construction and Anti-Cancer Activity of a Self-Assembly Composite Nano-Delivery System Loaded with Curcumin

**DOI:** 10.3390/molecules30142940

**Published:** 2025-07-11

**Authors:** Liang Chen, Qiao Wu, Chen Yang, Xiulan Xin, Zhaochu Xu, Shuai Luo, Hao Liang

**Affiliations:** 1 School of Bioengineering, Beijing Polytechnic University, Beijing 100176, China; chenliang@bpi.edu.cn (L.C.); yangchen2000521@126.com (C.Y.); xinxiulan@bpi.edu.cn (X.X.); xuzhaochu@bpi.edu.cn (Z.X.); luoshuai@bpi.edu.cn (S.L.); 2 State Key Laboratory of Chemical Resource Engineering, Beijing University of Chemical Technology, Beijing 100029, China; wuqiao@mail.buct.edu.cn

**Keywords:** natural self-assembled nano-delivery system, curcumin, hyaluronic acid, adenine, anti-cancer

## Abstract

Natural products possess potent pharmacological activities and health benefits. However, drawbacks such as water insolubility, poor stability, and low bioavailability limit their practical applications. This research is dedicated to the development of suitable natural self-assembled nano-delivery systems to encapsulate natural molecule drugs, improving their dispersion and stability in aqueous solution. As a model drug, curcumin (Cur) was encapsulated in zinc–adenine nanoparticles (Zn–Adenine), based on the self-assembly of a coordination matrix material. Hyaluronic acid (HA) was further functionalized on the surface of Cur@(Zn–Adenine) to realize a tumor-targeted delivery system. The morphology was characterized through TEM and zeta potential analyses, while the encapsulation mechanism of the nanoparticles was researched via XRD and FTIR. The formed Cur@(Zn–Adenine)@HA nanoparticles exhibited good drug loading efficiency and drug loading rate. Moreover, compared to free Cur, Cur-loaded (Zn–Adenine)@HA showed enhanced pH stability and thermal stability. In particular, Cur@(Zn–Adenine)@HA demonstrated excellent biocompatibility and strong specificity for targeting CD44 protein on cancer cells. The above results indicate that (Zn–Adenine)@HA NPs can serve as an effective nano-delivery system for hydrophobic substances.

## 1. Introduction

**Figure 1:**
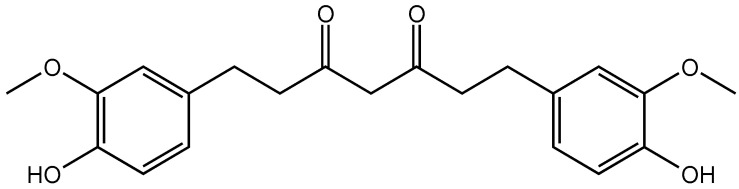
Structure of Cur.

Curcumin (Cur), a type of polyphenol, is extracted from the rootstalk of Zingiberaceae plants. Its chemical structure is shown in Figure 1. Its molecular formula is C_21_H_20_O_6_, and its molecular weight is 368.39 g/mol. Cur forms orange–yellow water insoluble crystals, but is soluble in glacial acetic acid, ethanol, methanol, acetone, and other organic reagents. It has strong coloring properties and is sensitive to light, heat, and other environmental factors [1,2].

Cur is a globally recognized natural pigment. It is generally applied in the food industry and has also been used as a natural treatment for cancer, inflammation, viral infections, and other diseases. Cur has low toxicity to normal cells but can induce the death of a variety of animal and human tumor cell lines, including lung, gastric, breast, colon, and prostatic cancers, and malignant melanoma [3,4]. Cur impedes the proliferation of tumor cells by suppressing multiple signal transduction pathways, disrupting cell cycles, and inducing apoptosis. Its molecular targets include NF-κB and AP-1 (activator protein 1), STAT3 (signal transducer and activator of transcription 3), and other carcinogenesis-related signaling proteins involved in the occurrence and development of cancer [5,6]. In a small-cell lung cancer model, Yang et al. found that Cur effectively reduced STAT3 phosphorylation, affecting cell proliferation, migration, invasion, apoptosis, and other behaviors, as well as altering the expression of proliferative proteins and prohibitin, thereby inhibiting tumor growth and angiogenesis [7]. However, its peculiarities, such as poor water insolubility, low stability, limited bioavailability, etc., have become major obstacles to its application in the medical field [8]. The advent of nanotechnology has helped to overcome these limitations.

Multifunctional drug delivery systems, enlightened by nanotechnology and high polymer chemistry—including lipid-based carriers (liposomes and lipid granules), polymer-based carriers (polymer nanospheres, micelle, and gelatum), and inorganic nanoparticles (gold and magnetic silicon dioxide)—are widely used in clinical medicine. These systems enhance drug absorption and distribution, improving therapeutic efficacy and drug safety [9]. Natural self-assembled drug delivery systems (NSADDS) are nano-carriers formed from natural biomaterials that spontaneously form into nanoscale structures such as inerratic spherical shapes, circular cylinders, or thin slices. These carriers exhibit enhanced penetration and retention and can be used to protect and transport active functional substances [10,11,12]. Compared with traditional embedding techniques, NSADDS offer size-dependent behaviors and specific biodistribution properties that benefit the targeted delivery of drugs and nutritious compounds [13,14]. Furthermore, biodegradable NSADDS increase the bioavailability of active molecules, enable the controlled and sustained release of guest molecules, and promote the targeted delivery of active molecules [15].

Metal biomolecule frameworks (MBioFs), built from biological components as single structural units and varying in modality and properties, have potential applications in separation and catalysis fields due to their high specific recognition and structural diversity [16]. Most importantly, based on their biological characteristics and biocompatibility, MBioFs are more suitable for drug dispensing and other medical applications. Along with developments in material science and pharmacology, the use of MBioFs composed of metal ions and nucleic acid components has become an important strategy for clinical cancer treatment and drug management due to their accessibility, biocompatibility, low cost, nanoscale size, high drug-loading efficiency, and self-assembly characteristics [17,18,19,20]. Adenine, a nucleic alkaline base that can be readily obtained from biomass, coordinates with multiple metal ions via its heterocycle and imidazole nitrogen atoms to form MBioFs, promoting its biological application potential [21,22,23,24]. Previous research has proven that zinc and adenine can self-assemble into porous materials suitable for drug storage and release, making zinc–adenine a preferred material for multifunctional drug delivery platforms [25]. Hyaluronic acid (HA), a major constituent of the human extracellular matrix, specifically binds the transmembrane receptor CD44 protein receptor, which is overexpressed on the surface of cancer cells. This property can be used for targeted tumor drug delivery [26,27,28]. Due to its biocompatibility, non-toxicity, and degradability in vivo, HA is also used as a controlled-release carrier for drugs [29,30,31].

In this study, we encapsulated Cur in zinc–adenine nanoparticles (Cur@(Zn–Adenine)NPs) and further functionalized (Cur@(Zn–Adenine)@HA NPs) the surface of Cur@Zn–Adenine with HA via electrostatic adsorption. Our aim was to build a tumor-targeted drug delivery system, providing an experimental and theoretical basis for targeted cancer therapy.

## 2. Results and Discussion

### 2.1. Preparation of Cur@(Zn-Adenine)@HA

Cur@(Zn–Adenine)@HA NPs were prepared using a one-pot process. In HEPES buffer solution, adenine coordinated with Zn^2+^ ions through its heterocycle and imidazole nitrogen atoms. During self-assembly, Cur was encapsulated within the MOF. HA was subsequently introduced to functionalize the surface of Cur@(Zn–Adenine), promoting cell uptake.

**Figure 2:**
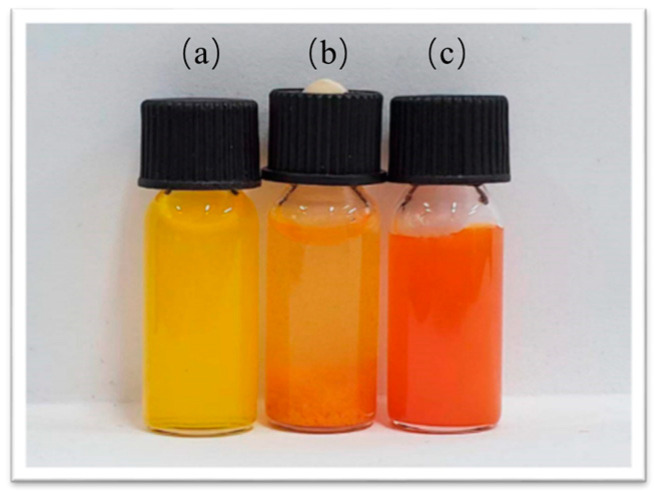
Photo of free Cur in water (**a**), Cur@(Zn-Adenine) (**b**), Cur@(Zn-Adenine)@HA (**c**).

As shown in Figure 2, the aqueous solution (a) of Cur appeared as a turbid, insoluble yellow liquid. Cur@(Zn–Adenine) (b) formed an aggregated orange precipitate. After HA modification, Cur@(Zn–Adenine)@HA (c) became a dispersed suspension with a slight color change.

### 2.2. DLE and DLC of Cur@(Zn-Adenine)@HA

**Figure 3:**
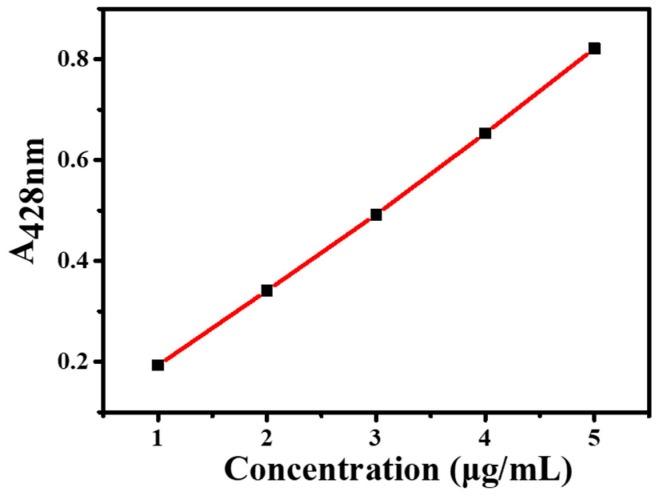
Standard curve of Cur.

The absorbance of Cur standard solutions was measured using UV–Vis spectroscopy. As shown in Figure 3, a standard curve was established with the following linear regression equation: y = 0.1568x + 0.0296 (R^2^ = 0.9993), where y represents absorbance and x denotes Cur concentration (μg/mL). The encapsulation efficiency of Cur@(Zn–Adenine) was 98.9 ± 1.24%. After HA modification, the drug loading capacity (DLC) of Cur@(Zn–Adenine)@HA reached 18.6 ± 0.65%. The results show that (Zn–Adenine)@HA is a highly suitable nanoplatform for delivering hydrophobic compounds.

### 2.3. Characterization of Cur@(Zn-Adenine)@HA

Cur@(Zn–Adenine), Cur@(Zn–Adenine)@HA, and (Zn–Adenine)@HA were characterized using TEM, grain diameter and electric potential, XRD, and FTIR. UV was also used to scan the full wavelength range of 200 nm–700 nm for the above sample.

**Figure 4:**
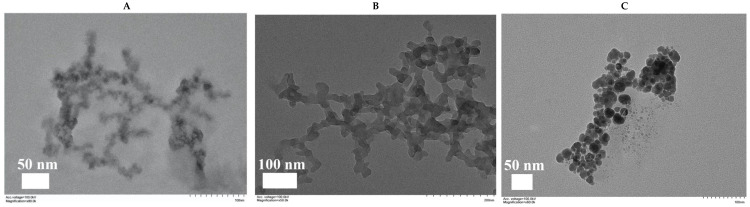
(**A**) TEM images of Zn-Adenine. (**B**) TEM images of Cur@(Zn-Adenine). (**C**) TEM images of Cur@(Zn-Ade nine)@HA.

Through TEM, we observed the nanostructure of Zn–Adenine, Cur@(Zn–Adenine), and Cur@(Zn–Adenine)@HA. As shown in Figure 4, Zn–Adenine exhibits an amorphous extended network structure that was easy to reunite. After Cur loading, the network structure expanded—consistent with its subsidence behavior in water. Compared with Cur@(Zn–Adenine), Cur@(Zn–Adenine)@HA presented a well-defined spherical structure with better dispersibility, which is beneficial for intravenous injection and to achieve the EPR effect [32].

**Figure 5:**
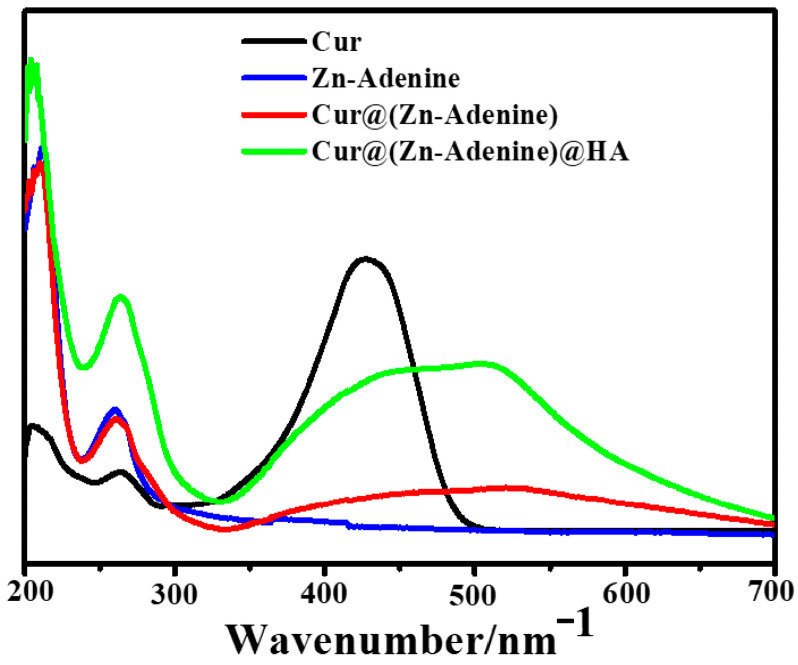
The UV spectra of Cur, Zn-Adenine, Cur@(Zn-Adenine), and Cur@(Zn-Adenine)@HA.

The optical properties of NPs were recorded using UV spectra. As shown in Figure 5, Cur@(Zn–Adenine) displays a UV–visible absorption pattern combining Cur and Zn–Adenine. Cur@(Zn–Adenine)@HA showed a similar ultraviolet absorption trend to Cur@(Zn–Adenine). The UV absorption peak of free Cur at 427 mm red-shifted to 520 nm and 510 nm in the absorption spectrum of Cur@(Zn–Adenine) and Cur@(Zn–Adenine)@HA, respectively. These results indicate that Cur was successfully enveloped in the Zn–Adenine and (Zn–Adenine)@HA NPs, and that this envelopment partially altered the UV absorption of Cur [33].

**Figure 6:**
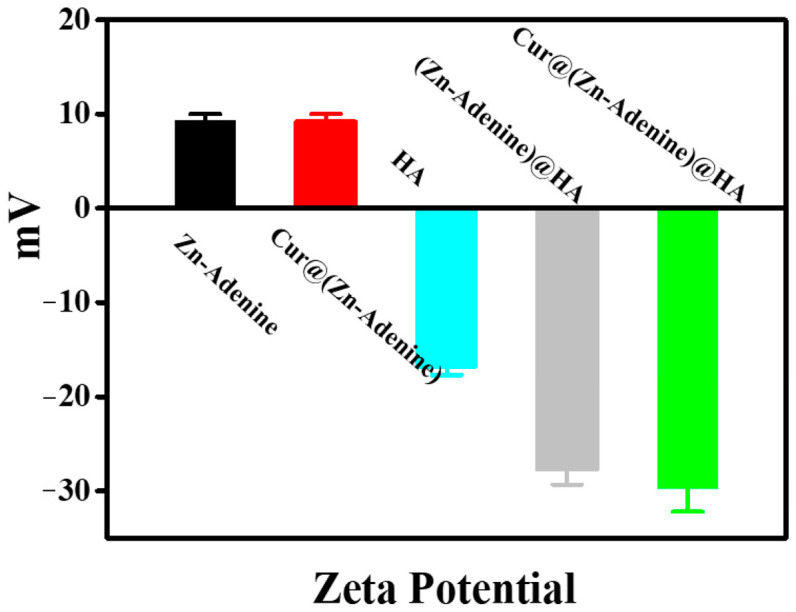
The zeta potentials of Zn-Adenine, Cur@(Zn-Adenine), HA, (Zn-Adenine)@HA, and Cur@(Zn-Adenine)@HA.

In addition, the surface potential of nanoparticles was measured. As shown in Figure 6, the HA surface was negatively charged (−16.97 mV), and the zeta electric potential of Cur@(Zn–Adenine) (9.20 mV) was almost the same as that of Zn–Adenine (9.27 mV). The zeta electric potential of Cur@(Zn–Adenine)@HA and (Zn–Adenine)@HA was significantly reduced to −29.8 mV and −27.7 mV, respectively. Zeta potential was employed to assess the colloidal stability of the nanoparticles. The study revealed a significant increase in the magnitude of zeta potential following HA modification, indicating enhanced nanoparticle stability and improved resistance to sedimentation in solution.

**Figure 7:**
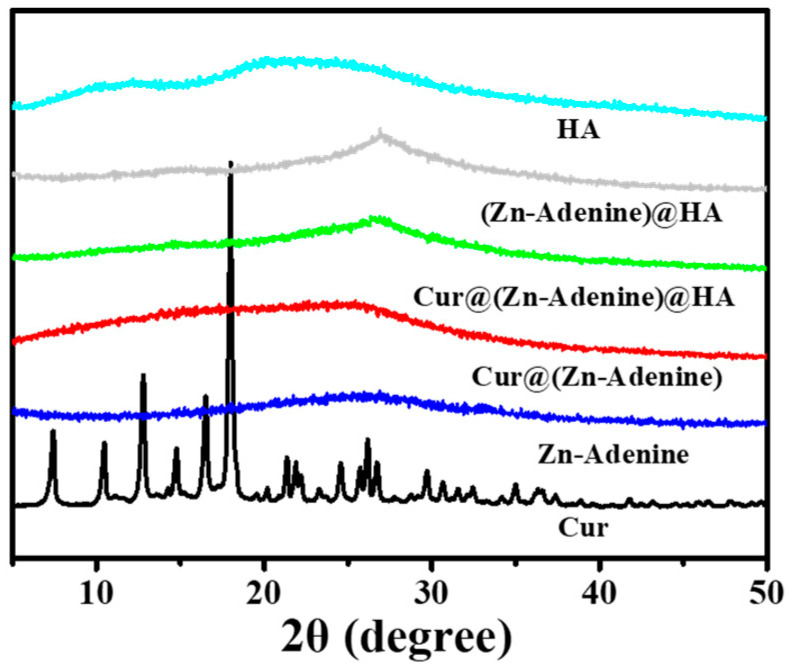
The XRD patterns of Cur, Zn-Adenine, Cur@(Zn-Adenine), Cur@(Zn-Adenine)@HA, (Zn-Adenine)@HA, and HA.

XRD was used to verify the successful structuring of the nanoplatform. As shown in Figure 7, Cur@(Zn–Adenine) closely matched the amorphous state of Zn–Adenine, and the crystalline peak of Cur was completely covered. This indicated that Cur was enveloped in Zn–Adenine, and that drug loading did not destroy the structural integrity of the nanoparticles. (Zn–Adenine)@HA and Cur@(Zn–Adenine)@HA showed different XRD patterns compared to Zn–Adenine, Cur@(Zn–Adenine), and HA, due to their good dispersibility [34]. On the other hand, TEM detected that after HA modification, the appearance of Cur@(Zn–Adenine) changed to a nearly spherical shape, which also contributed to the changes observed in XRD [35].

**Figure 8:**
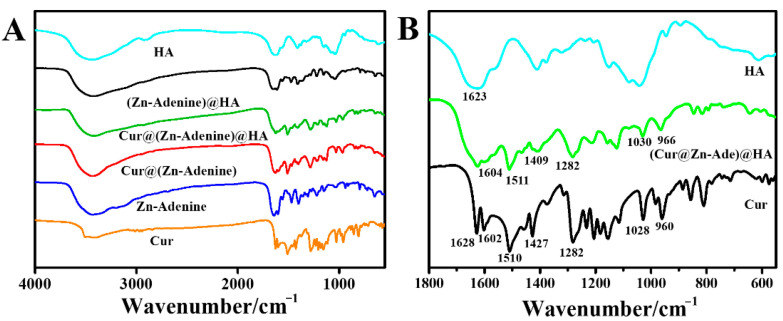
(**A**) FT-IR spectra of Cur, Zn-Adenine, Cur@(Zn-Adenine), Cur@(Zn-Adenine)@HA, (Zn-Adenine)@HA, and HA. (**B**) the enlarged FT-IR spectra of Cur, Cur@(Zn-Adenine)@HA and HA in the range 1800–550 cm^−1^.

Figure 8A presents the FTIR spectrum of Cur and NPs between 4000 and 400 cm^−1^, and Figure 8B shows an enlarged view of 1800–550 cm^−1^. In Cur@(Zn–Adenine)@HA, the characteristic peaks of Cur at 1628 (C=O stretching) and 1602 cm^−1^ (aromatic series C=C stretching) disappear. The peaks of Cur located at 1510 (C=O stretching), 1427 (olefin bending vibration bonded by C-H and benzene ring), and at 1028 and 960 cm^−1^ (C=O stretching) shift separately to 1511, 1409, 1030, and 966 cm^−1^ in the Cur@(Zn–Adenine)@HA optical spectrum. It is speculated that the benzene ring structure in Cur undergoes π-π stacking with the aromatic ring structure in Adenine, slightly altering the vibration peak displacement on the benzene ring of Cur [36,37]. Moreover, the peak located at 1623 cm^−1^ in the amide I band of HA corresponds to the peak located at 1604 cm^−1^ in the Cur@(Zn–Adenine)@HA spectrum, which corresponds to changes caused by asymmetric tensile vibration of the -COO anion [38]. These results further confirmed that Cur was loaded into Zn–Adenine and that HA was successfully modified onto the surface of Cur@(Zn–Adenine).

### 2.4. Stability of Cur@(Zn-Adenine)@HA

**Figure 9:**
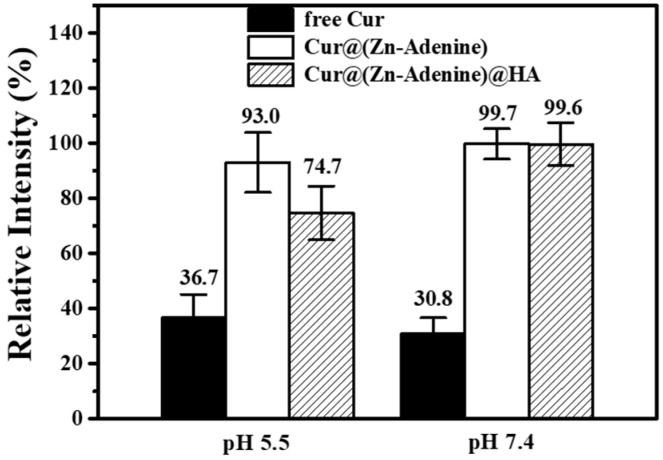
Stability of free and NPs loaded Cur in PBS solution (pH 5.5 or 7.4) after 120 min.

At different pH values, and with all other test and commissioning conditions kept the same, the photophobic reaction was carried out for 120 min. The relative intensity of free Cur, Cur@(Zn–Adenine), and Cur@(Zn–Adenine)@HA in PBS solution (10 mM, 25 °C) is shown in Figure 9. After reacting for 120 min in the solution at pH 5.5 and 7.4, the Cur content dropped to below 40%. By contrast, Cur@(Zn–Adenine) showed a loss of less than 10% in both solutions, while Cur@(Zn–Adenine)@HA experienced a content loss of approximately 25.3% in the pH = 5.5 solution.

**Figure 10:**
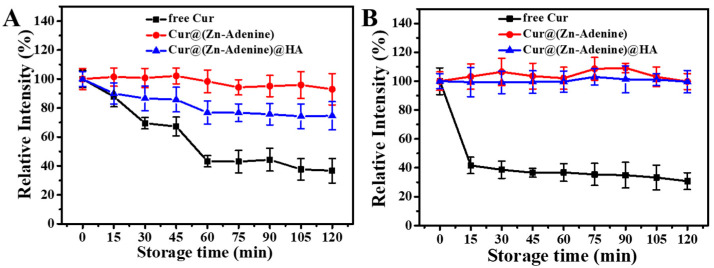
(**A**) Degradation kinetics of free and NPs loaded Cur in PBS solution (pH 5.5) for 120 min. (**B**) Degradation kinetics of free and NPs loaded Cur in PBS solution (pH 7.4) for 120 min.

Subsequently, we studied the degradation kinetics of free and enveloped Cur in aqueous solution. As shown in Figure 10A, in PBS solution at pH = 5.5, the relative intensity of free Cur and Cur-loaded NPs gradually decreased over time. Only 49.19% of free Cur remained after 60 min, then gradually degraded, with relative intensity dropping to 36.69% after 120 min. Cur in Cur@(Zn–Adenine) still retained above 90% after 120 min, while Cur in Cur@(Zn–Adenine)@HA degraded 23.64% after 60 min and then remained stable. As shown in Figure 10B, in PBS solution with pH = 7.4, the relative intensity of free Cur rapidly decreased to 41.63% within 15 min. By contrast, Cur in Cur@(Zn–Adenine) and Cur@(Zn–Adenine)@HA showed almost no degradation within 120 min. The results showed that the envelopment of Zn–Adenine and (Zn–Adenine)@HA could both significantly enhance the pH stability of Cur, but the effect of (Zn–Adenine)@HA was slightly weaker, possibly because the modification of HA made the NPs more dispersed in aqueous solution, increasing their contact with the external environment and, hence, weakening stability.

**Figure 11:**
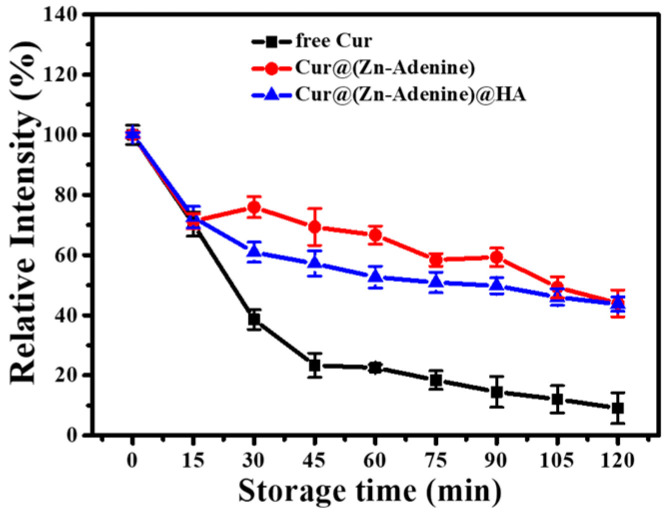
Degradation kinetics of free and NPs loaded Cur in PBS solution (pH 7.4) at 80 °C for 120 min.

During heat treatment at 80 °C, degradation kinetics curves of free Cur and NP-loaded Cur are shown in Figure 11. Free Cur showed rapid degradation within 45 min, rapidly reducing to 23.34%, and then degraded slowly, with the final relative intensity reduced to 9.12% after 120 min. Cur loaded by Zn–Adenine and (Zn–Adenine)@HA showed a rapid degradation process within 15 min, then degraded more slowly. After heat treatment for 120 min, the stability of Cur was increased more than four times. This showed that the envelopment of Zn–Adenine and (Zn–Adenine)@HA could improve the heat stability of Cur.

### 2.5. Cellular Uptake of Cur@(Zn-Adenine)@HA

**Figure 12:**
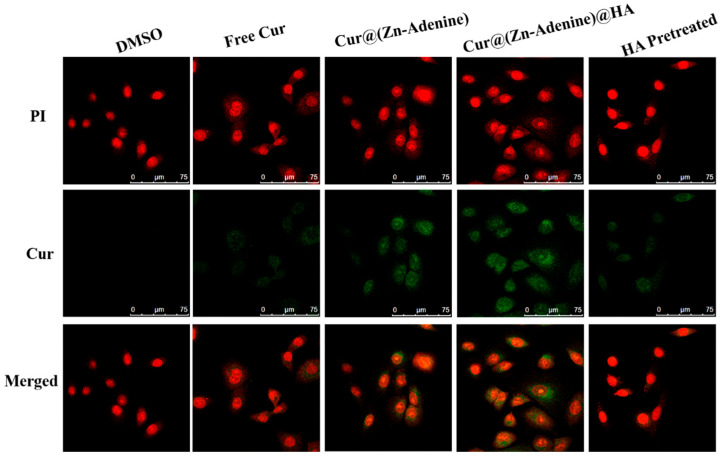
CLSM images on A549 cells incubated with DMSO, free Cur, Cur@(Zn-Adenine), Cur@(Zn-Adenine)@HA, and Cur@(Zn-Adenine)@HA after HA pretreated.

Due to its specific recognition with the CD44 receptor on the tumor cell surface, HA has been widely researched and applied to the field of targeted antitumor drugs. The uptake of free Cur and Cur-loaded NPs by A549 cells was explored through CLSM. Commercial fluorescent dye PI (red) was used to characterize cell morphology, and the green fluorescence of Cur indicated cellular uptake of the NPs. A Cur concentration of 5 μg/mL was selected as the final equivalent administration concentration. DMSO-treated cells were used as the control, and Cur, Cur@(Zn–Adenine), and Cur@(Zn–Adenine)@HA were used separately to treat monolayer cells for 4 h. In order to verify the HA targeting mechanism, competitive treatment with excess HA was performed—that is, free HA was used to preprocess cells and then had uptake of Cur@(Zn–Adenine)@HA. As shown in Figure 12, cells treated with free Cur showed weak green fluorescence, indicating a low uptake of Cur. Cells treated with Cur@(Zn–Adenine) and Cur@(Zn–Adenine)@HA showed stronger Cur fluorescence, and HA-modified NPs showed brighter fluorescence and wider distribution. This showed that the modification with Zn–Adenine and HA could promote the absorption of NPs by cells and that HA’s targeting of cancer cells could promote drug endocytosis. After A549 cells were pretreated with HA, the fluorescence signal of Cur@(Zn–Adenine)@HA in the cells was significantly reduced, indicating a specific competitive relationship between Cur@(Zn–Adenine)@HA and HA for the CD44 receptor overexpressed on the cell surface.

**Figure 13:**
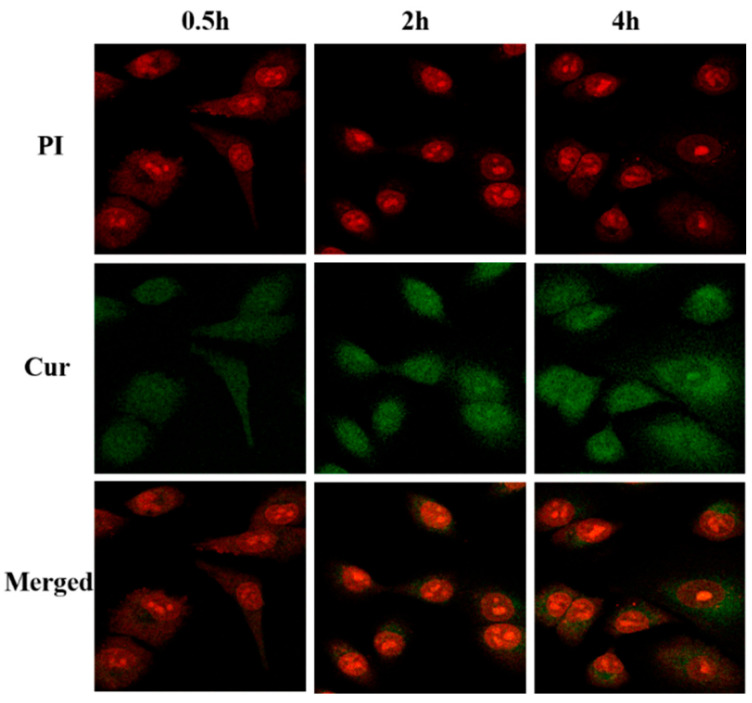
CLSM images on A549 cells incubated with Cur@(Zn-Adenine)@HA for 0.5 h, 2 h, and 4 h.

Figure 13 shows a CLSM diagram of cells treated with Cur@(Zn–Adenine)@HA for 0.5 h, 2 h, and 4 h, and as time went on, the green fluorescence intensity in the cells gradually increased, indicating that the release behavior of Cur in A549 cells was time-dependent. This durative release is the basis for prolonged drug therapy. In short, the results showed that Cur@(Zn–Adenine)@HA could effectively increase the accumulation of Cur in cells and enhance the therapeutic effect through tumor cell-targeted drug endocytosis between HA and the CD44 receptor.

### 2.6. Cytotoxicity of Cur@(Zn-Adenine)@HA

**Figure 14:**
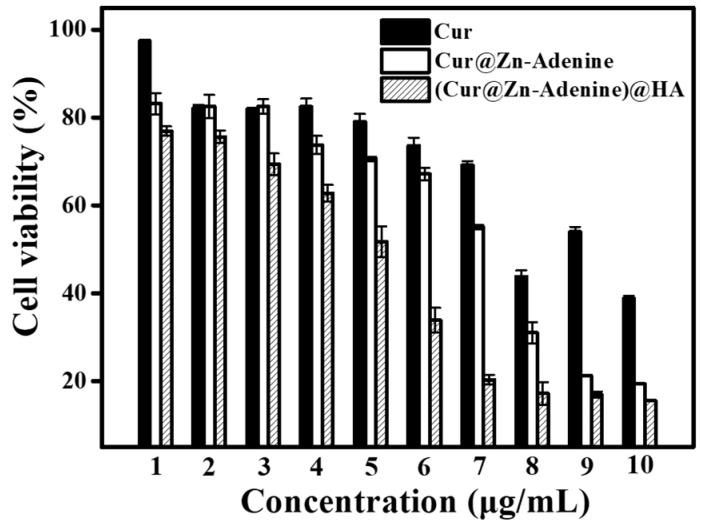
Anti-proliferation activity free Cur, Cur@(Zn-Adenine) and Cur@(Zn-Adenine)@HA against A549 cells.

Through a quantitative cytotoxicity method, the inhibitory effects of free Cur, Cur@(Zn–Adenine), and Cur@(Zn–Adenine)@HA on A549 cells were studied under the same effective Cur concentration. As shown in Figure 14, with increasing Cur concentration, the cell survival rates of free Cur, Cur@(Zn–Adenine), and Cur@(Zn–Adenine)@HA gradually declined, indicating that both free Cur and Cur-loaded NPs had an inhibitory effect on tumor cells. When the Cur concentration was 1 μg/mL, the cell survival rate of free Cur was 97.32%, that of Cur@(Zn–Adenine) was 83.18%, and that of Cur@(Zn–Adenine)@HA was 76.92%. At a Cur concentration of 7 μg/mL, free Cur induced only 30.83% cell death, Cur@(Zn–Adenine) induced 44.94% cell death, and Cur@(Zn–Adenine)@HA induced 79.69% cell death, with its cancer cell suppression effect increasing about 2.58 times compared to free Cur. When the concentration reached 10 μg/mL, free Cur caused a 61.21% cell death rate, Cur@(Zn–Adenine) 80.65%, and Cur@(Zn–Adenine)@HA as high as 84.5%. The results showed that Cur@(Zn–Adenine) could increase the bioavailability of Cur, and after HA modification, its anticancer effect was significantly increased, which could be attributed to improved stability. Cur showed massive degradation in the weakly acidic tumor environment, while the enveloping carrier enabled effective and continuous release. On the other hand, the targeting effect of HA on cancer cells promoted drug endocytosis of drugs and enabled cumulative drug release in the cells.

### 2.7. Live/Dead Cell Staining Assay

**Figure 15:**
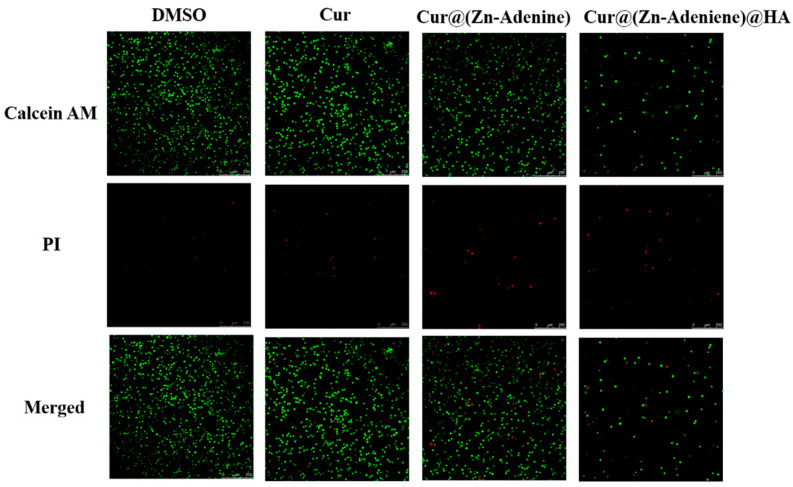
CLSM images of Calcein AM and PI stained A549 cells treated with DMSO, Cur, Cur@(Zn-Adenine), and Cur@(Zn-Adenine)@HA. Scale bar = 250 µm.

The CLSM observation of NPs’ inhibitory effect on cancer cells was assisted by a cell death and survival experiment. In this experiment, Calcein-AM marked live cells with green fluorescence, while PI stained dead cells with red. As shown in Figure 15, DMSO and free Cur showed almost no toxicity to A549 cells, and all the cells were in good condition. Cell death was observed in cells treated with both Cur@(Zn–Adenine) and Cur@(Zn–Adenine)@HA. However, the limited cytotoxic effect was likely due to the abbreviated 5-hour treatment duration, during which the cumulative release of encapsulated Cur in Zn–Adenine and (Zn–Adenine)@HA carriers remained insufficient to achieve complete tumor cell eradication.

### 2.8. Cancer Cell Targeting of Cur@(Zn-Adenine)@HA

**Figure 16:**
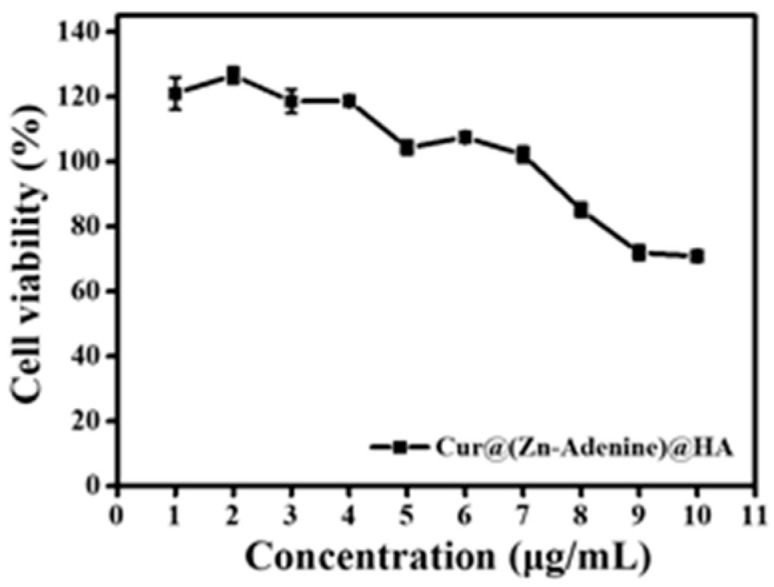
Anti-proliferation activity of Cur@(Zn-Adenine)@HA after HA pre-incubated with A549 cells.

The cancer cell targeting of HA was based on the specific recognition between HA and the CD44 protein receptor overexpressed on the surface of cancer cells. The interaction between HA and the CD44 receptor on the surface of A549 cells was verified through experiments. After incubating A549 cells with HA solution for 12 h, cells were treated at Cur@(Zn–Adenine)@HA concentrations of 1–10 μg/mL. As shown in Figure 16, when the Cur concentration was 1–7 μg/mL, the inhibition rate of Cur@(Zn–Adenine)@HA on A549 cells was almost negligible. When the Cur concentration rose from 8 to 10 μg/mL, the cell survival rate was still above 70%. This finding showed that after competitive inhibition of HA, the targeting specificity of Cur@(Zn–Adenine)@HA, mediated by HA, weakened. As a result, its recognition of tumor cells was reduced, and the endocytosis of the drug by cells was affected. The above results demonstrated that the surface modification role of HA remarkably improved the cellular uptake and anticancer effect of NPs.

## 3. Materials and Methods

### 3.1. Materials

Cur and HA were purchased from Meilunbio (Dalian, China). Adenine, Zn(NO_3_)_2_·6H_2_O, and HEPES buffer solution were obtained from Aladdin (Shanghai, China). Ethanol and acetonitrile were purchased from Mcklin (Shanghai, China).

PBS (pH = 5.5, 7.4), DMEM, DMSO, and FBS were obtained from Servicebio (Wuhan, China). A549 cells (glandular cancer human alveolus base epithelial cells) were obtained from the Cell Resource Center of Peking Union Medical College.

### 3.2. Preparation of Cur@(Zn-Adenine)@HA

Cur@(Zn–Adenine)@HA NPs were prepared using a one-pot process. Cur ethanol solution (2 mg/mL Cur), HEPES buffer solution (50 mM, pH = 7.4), Zn(NO_3_)_2_·6H_2_O water solution (50 mM), adenine water solution (10 mM), and HA water solution (1 mg/mL) were prepared separately. Then, 4 mL of adenine water solution, 4 mL of Cur ethanol solution, and 4 mL of Zn(NO_3_)_2_·6H_2_O water solution were added sequentially into 20 mL of HEPES buffer solution, followed by intense agitation (800 rpm/min) of the mixture in the dark for 2 h. The suspension was centrifuged, and the precipitate at the bottom was washed with deionized water three times to obtain Cur@(Zn–Adenine) NPs, which were used either in the next reaction or freeze-dried for backup. Zn–Adenine NPs were prepared using the same method. The collected Cur@(Zn–Adenine) NPs were dispersed in 5 mL of deionized water, then poured into a small beaker filled with 20 mL of HA solution. The mixture was stirred vigorously for 4 h. After centrifugation for 30 min to remove unlinked HA, the precipitate at the bottom was washed with deionized water three times to obtain Cur@(Zn–Adenine)@HA NPs.

### 3.3. DLE and DLC of Cur@(Zn-Adenine)@HA

A 1 mg/mL Cur solution in ethanol was stepwise diluted to concentrations of 1, 2, 3, 4, and 5 μg/mL. The Cur standard solutions were then analyzed by UV–Vis spectroscopy at a detection wavelength of 428 nm to establish a standard curve.

A weighed amount of Cur-loaded NPs powder was dissolved in ethanol solution, sonicated in a water bath for 30 min, then further diluted to a suitable concentration with ethanol solution, and tested for Cur content through UV. The Cur content at the initial time was set to 100%. The embedding rate and drug loading rate were calculated according to the Cur standard curve and Equations (1) and (2).

Embedding rate (%) = Content of Cur in Cur@(Zn-Adenine)/ total amount of Cur added × 100%(1)

Drug loading rate (%) = Content of Cur in Cur@(Zn-Adenine)@HA/total amount of nano particles added × 100%(2)

### 3.4. Characterization of Cur@(Zn-Adenine)@HA

Solutions of Zn–Adenine, Cur@(Zn–Adenine), and Cur@(Zn–Adenine)@HA were diluted to appropriate concentrations, minimally spotted onto ultrathin silicon wafers/400-mesh copper grids, and air-dried at room temperature. Morphological characterization was performed using transmission electron microscopy (TEM).

HA, Zn–Adenine, Cur@(Zn–Adenine), Cur@(Zn–Adenine)@HA, and (Zn–Adenine)@HA solutions were equally diluted under identical conditions. Surface zeta potential was determined using a dynamic light scattering instrument equipped with zeta potential measurement capability.

Appropriate amounts of dried powders of HA, Zn–Adenine, Cur@(Zn–Adenine), Cur@(Zn–Adenine)@HA, and (Zn–Adenine)@HA were evenly spread on clean sample holders. Structural analysis was conducted via X-ray diffraction (XRD) with a scan rate of 10°/min over a 2θ range of 5°–40° and a step size of 0.02°.

### 3.5. Stability of Cur@(Zn-Adenine)@HA

The influence of temperature (80 °C) and pH (7.4, 5.5) on NPs stability was examined. For temperature stability, Cur@(Zn–Adenine) and Cur@(Zn–Adenine)@HA of equal Cur concentration were dispersed in PBS buffer solution (pH = 7.4, 10 mM), and a Cur solution of the same concentration (PBS, pH = 7.4, 10 mM) served as the control. For the stability of NPs in a pH = 5.5 solution, the buffer solution of 10 mM, pH = 7.4 was replaced with a PBS of 10 mM, pH = 5.5. The test duration was 2 h.

### 3.6. Cell Lines and Cell Culture

A549 cells (glandular cancer human alveolus base epithelial cells) were cultured in DMEM containing 10% fetal calf serum (FBS) and 1% double-resistance (100 U/mL streptomycin and 100 U/mL penicillin). Incubator conditions: 37 °C, 5% CO_2_.

### 3.7. Cellular Uptake of Cur@(Zn-Adenine)@HA

A549 cells were seeded in glass-bottom confocal dishes at a density of 40,000 cells per mL (200 μL per dish) and incubated for 12 h. Solutions of Cur, Cur@(Zn–Adenine), and Cur@(Zn–Adenine)@HA were diluted with culture medium to achieve a final Cur concentration of 5 μg/mL. Cells were then treated with these solutions and co-incubated for 4 h. CLSM images of A549 were acquired to observe cellular uptake of Cur, Cur@(Zn–Adenine), and Cur@(Zn-Adenine)@HA. The selected Cur final concentration was 5 μg/mL; cells were incubated for 4 h, and captured laser confocal microscope (CLSM) images of A549 cells for Cur, Cur@(Zn–Adenine), and Cur@(Zn–Adenine)@HA were captured. Particularly, CLSM images of cells and Cur@(Zn–Adenine)@HA were also captured after 0.5 h, 2 h, and 4 h to assess changes in cell uptake over time. For inhibition studies, A549 cells were pretreated with 2 mg/mL free HA for 4 h, then Cur@(Zn–Adenine)@HA was incubated with the cells for 4 h. CLSM images were taken to observe the influence of HA inhibition on the cellular uptake of Cur@(Zn–Adenine)@HA.

### 3.8. Cytotoxicity of Cur@(Zn-Adenine)@HA

The cytotoxicity of Cur@(Zn–Adenine)@HA against A549 cells was evaluated using a CCK-8 kit and compared with free Cur and Cur@(Zn–Adenine). A549 cells in the logarithmic growth phase were trypsinized, centrifuged, and adjusted to a density of 4000 cells/mL. Cells were seeded into 96-well plates and incubated for 12 h to allow attachment. Subsequently, cells were treated with Zn-Adenine or (Zn–Adenine)@HA across a concentration range of 0–100 μg/mL. After treatment, CCK-8 reagent diluted in culture medium (10 μL per well) was added to each well, followed by incubation in the dark for 1 h. Absorbance was measured at 450 nm using a microplate reader to determine cell viability.

### 3.9. Live/Dead Cell Staining Assay

A549 cells were inoculated into laser confocal wells at 5 × 10^5^ cells/well and incubated for 24 h. After removing the medium, each well received 100 μL of solution (Cur concentration: 5 μg/mL), pre-dissolved in DMSO and diluted with DMEM, was added to each well for Cur, Cur@(Zn–Adenine), and Cur@(Zn–Adenine)@HA and cultured for 5 h. The control group included cells only treated with DMSO. All the experimental groups and control groups were washed once with PBS, followed by the addition of Calcein-AM/PI in PBS solution. Samples were incubated at 37 °C for 15 min. Each hole was fully washed 3 times with PBS and kept away from light before CLSM observation.

### 3.10. Cancer Cell Targeting of Cur@(Zn-Adenine)@HA

After preprocessing A549 cells with HA (2 mg/mL) for 12 h, Cur@(Zn–Adenine)@HA with a concentration range of 1–10 μg/mL was added to incubate for 72 h. The CCK-8 test kit was used to test cell survival rate and evaluate cell viability.

### 3.11. Statistical Analysis

All experiments were performed in triplicate. Data are expressed as X ± SD, and variance analysis was performed using Origin 9.

## 4. Conclusions

In this study, a Bio-MOF nano drug-loading system based on Zn–Adenine was successfully prepared, and Cur loading was carried out. In addition, HA with specific recognition characteristics for cancer cells was modified on its surface, forming a targeted delivery system, Cur@(Zn–Adenine)@HA NPs. Cur@(Zn–Adenine)@HA NPs showed a high embedding rate (98.9 ± 1.24%) and high drug loading rate (18.6 ± 0.65%) and were well-dispersed in aqueous solution, thus improving the subsidence behavior of the original MOF material. Their structure and formation mechanism were explored through various means of characterization. Cur@(Zn–Adenine)@HA NPs significantly improved the heat stability of the embedded drug Cur and increased its stability in weakly acidic tumor environments (pH = 5.5) by more than two times. Its stability in physiological pH increased by about 3.2 times, helping to protect the loss of encapsulated drug due to degradation during transportation before reaching tumor cells. Compared with the equivalent dose of free Cur or Cur@(Zn–Adenine), Cur@(Zn-Adenine)@HA NPs more effectively reduced the survival rate of A549 cells. When Cur concentration was 7 μg/mL, its cancer cell suppression effect increased about 2.58 times compared with free Cur. Furthermore, the research results showed that Cur@(Zn–Adenine)@HA NPs achieved targeted delivery and controlled release of Cur through the specific binding of HA to the highly expressed receptor CD44 on the surface of cancer cells. This promoted drug endocytosis and accumulation in the cells while reducing harm to normal cells. In summary, the (Zn-Adenine)@HA NPs constructed in this study facilitate the endocytosis of A549 cells through CD44 receptor-specific interactions, enabling targeted delivery and controlled release of hydrophobic sensitive drugs while enhancing their stability under physiological conditions, demonstrating great potential as a versatile drug delivery platform. However, in order to apply them and explore their impact on human health, follow-up studies on (Zn–Adenine)@HA NPs are still required. These include investigating the bioactivity of the nanoplatform’s encapsulated molecules after body fluid circulation, their release mechanism in the human body, and their transmission behavior and absorption mechanism in animal systems, with the expectation of promoting their future application in the medical field.

## Data Availability

The data presented in this study are available on request from the corresponding author.

## References

[1] Perrone D., Ardito F., Giannatempo G., Dioguardi M., Troiano G., Lo R.L., DE Lillo A., Laino L., Lo M.L. (2015). Biological and therapeutic activities, and anticancer properties of curcumin. Exp. Ther. Med..

[2] Li S., Fang C., Zhang J., Liu B., Wei Z., Fan X., Sui Z., Tan Q. (2016). Catanionic lipid nanosystems improve pharmacokinetics and anti-lung cancer activity of curcumin. Nanomedicine.

[3] Elmegeed G.A., Yahya S.M., Abd-Elhalim M.M., Mohamed M.S., Mohareb R.M., Elsayed G.H. (2016). Evaluation of heterocyclic steroids and curcumin derivatives as anti-breast cancer agents: Studying the effect on apoptosis in MCF-7 breast cancer cells. Steroids.

[4] Yallapu M.M., Khan S., Maher D.M., Ebeling M.C., Sundram V., Chauhan N., Ganju A., Balakrishna S., Gupta B.K., Zafar N. (2014). Anti-cancer activity of curcumin loaded nanoparticles in prostate cancer. Biomaterials.

[5] Lopez-Lazaro M. (2008). Anticancer and carcinogenic properties of curcumin: Considerations for its clinical development as a cancer chemopreventive and chemotherapeutic agent. Mol. Nutr. Food Res..

[6] Shehzad A., Wahid F., Lee Y.S. (2010). Curcumin in cancer chemoprevention: Molecular targets, pharmacokinetics, bioavailability, and clinical trials. Arch. Pharm..

[7] Yang C.L., Liu Y.Y., Ma Y.G., Xue Y.X., Liu D.G., Ren Y., Liu X.B., Li Y., Li Z. (2012). Curcumin blocks small cell lung cancer cells migration, invasion, angiogenesis, cell cycle and neoplasia through Janus kinase-STAT3 signalling pathway. PLoS ONE.

[8] Subramanian A.P., Jaganathan S.K., Manikandan A., Pandiaraj K.N., Gomathi N., Supriyanto E. (2016). Recent trends in nano-based drug delivery systems for efficient delivery of phytochemicals in chemotherapy. RSC Adv..

[9] Cui W., Li J., Decher G. (2016). Self-Assembled smart nanocarriers for targeted drug delivery. Adv. Mater..

[10] Duan X., Chen H., Fan L., Kong J. (2016). Drug Self-Assembled delivery system with dual responsiveness for cancer chemotherapy. ACS Biomater. Sci. Eng..

[11] Li Y., Wang Y., Huang G., Gao J. (2018). Cooperativity principles in Self-Assembled nanomedicine. Chem. Rev..

[12] Jin Y., Xin R., Ai P., Chen D. (2008). Self-assembled drug delivery systems 2. Cholesteryl derivatives of antiviral nucleoside analogues: Synthesis, properties and the vesicle formation. Int. J. Pharm..

[13] Qin S.Y., Zhang A.Q., Cheng S.X., Rong L., Zhang X.Z. (2017). Drug self-delivery systems for cancer therapy. Biomaterials.

[14] Jaiswal S., Mishra P. (2018). Co-delivery of curcumin and serratiopeptidase in HeLa and MCF-7 cells through nanoparticles show improved anti-cancer activity. Mater. Sci. Eng. C Mater. Biol. Appl..

[15] Lu H., Wang Q., Li G., Qiu Y., Wei Q. (2017). Electrospun water-stable zein/ethyl cellulose composite nanofiber and its drug release properties. Mater. Sci. Eng. C Mater. Biol. Appl..

[16] Wu H., Tian C., Zhang Y., Yang C., Zhang S., Jiang Z. (2015). Stereoselective assembly of amino acid-based metal-biomolecule nanofibers. Chem. Commun..

[17] Pu F., Ren J., Qu X. (2018). Nucleobases, nucleosides, and nucleotides: Versatile biomolecules for generating functional nanomaterials. Chem. Soc. Rev..

[18] Joarder B., Chaudhari A.K., Nagarkar S.S., Manna B., Ghosh S.K. (2013). Amino acid based dynamic metal-biomolecule frameworks. Chemistry.

[19] Liang H., Lin F., Zhang Z., Liu B., Jiang S., Yuan Q., Liu J. (2017). Multicopper laccase mimicking nanozymes with nucleotides as ligands. ACS Appl. Mater. Interfaces.

[20] Lippert B., Sanz M.P. (2016). The renaissance of Metal-Pyrimidine nucleobase coordination chemistry. Acc. Chem. Res..

[21] Mohapatra B., Pratibha, Verma S. (2017). Directed adenine functionalization for creating complex architectures for material and biological applications. Chem. Commun..

[22] An J., Geib S.J., Rosi N.L. (2009). Cation-triggered drug release from a porous zinc-adeninate metal-organic framework. J. Am. Chem. Soc..

[23] Sushrutha S.R., Hota R., Natarajan S. (2016). Adenine-Based coordination polymers: Synthesis, structure, and properties. Eur. J. Inorg. Chem..

[24] Purohit C.S., Verma S. (2006). A luminescent silver-adenine metallamacrocyclic quartet. J. Am. Chem. Soc..

[25] An J., Farha O.K., Hupp J.T., Pohl E., Yeh J.I., Rosi N.L. (2012). Metal-adeninate vertices for the construction of an exceptionally porous metal-organic framework. Nat. Commun..

[26] Luan S., Zhu Y., Wu X., Wang Y., Liang F., Song S. (2017). Hyaluronic-Acid-Based pH-Sensitive nanogels for Tumor-Targeted drug delivery. ACS Biomater. Sci. Eng..

[27] Huang G., Huang H. (2018). Hyaluronic acid-based biopharmaceutical delivery and tumor-targeted drug delivery system. J. Control. Release.

[28] Wolf K.J., Kumar S. (2019). Hyaluronic Acid: Incorporating the Bio into the Material. ACS Biomater. Sci. Eng..

[29] Xu W., Qian J., Hou G., Wang Y., Wang J., Sun T., Ji L., Suo A., Yao Y. (2019). A dual-targeted hyaluronic acid-gold nanorod platform with triple-stimuli responsiveness for photodynamic/photothermal therapy of breast cancer. Acta Biomater..

[30] Spadea A., Rios D.L.R.J., Tirella A., Ashford M.B., Williams K.J., Stratford I.J., Tirelli N., Mehibel M. (2019). Evaluating the efficiency of hyaluronic acid for tumor targeting via CD44. Mol. Pharm..

[31] Ossipov D.A. (2010). Nanostructured hyaluronic acid-based materials for active delivery to cancer. Expert Opin. Drug Deliv..

[32] Zhang H., Li Q., Liu R., Zhang X., Li Z., Luan Y. (2018). A versatile prodrug strategy to in situ encapsulate drugs in MOF nanocarriers: A case of Cytarabine-IR820 prodrug encapsulated ZIF-8 toward Chemo-Photothermal therapy. Adv. Funct. Mater..

[33] Song L., Pan Z., Zhang H., Li Y., Zhang Y., Lin J., Su G., Ye S., Xie L., Li Y. (2017). Dually folate/CD44 receptor-targeted self-assembled hyaluronic acid nanoparticles for dual-drug delivery and combination cancer therapy. J. Mater. Chem. B.

[34] Shi Z., Chen X., Zhang L., Ding S., Wang X., Lei Q., Fang W. (2018). FA-PEG decorated MOF nanoparticles as a targeted drug delivery system for controlled release of an autophagy inhibitor. Biomater. Sci..

[35] Jing Y., Wang J., Yu B., Lun J., Cheng Y., Xiong B., Lei Q., Yang Y., Chen L., Zhao M. (2017). A MOF-derived ZIF-8@Zn1-xNixO photocatalyst with enhanced photocatalytic activity. RSC Adv..

[36] Zhang X., Liu L., Huang L., Zhang W., Wang R., Yue T., Sun J., Li G., Wang J. (2019). The highly efficient elimination of intracellular bacteria via a metal organic framework (MOF)-based three-in-one delivery system. Nanoscale.

[37] Govindaraj P., Kandasubramanian B., Kodam K.M. (2014). Molecular interactions and antimicrobial activity of curcumin (Curcuma longa) loaded polyacrylonitrile films. Mater. Chem. Phys..

[38] Cai W., Gao H., Chu C., Wang X., Wang J., Zhang P., Lin G., Li W., Liu G., Chen X. (2017). Engineering phototheranostic nanoscale Metal-Organic frameworks for multimodal Imaging-Guided cancer therapy. ACS Appl. Mater. Interfaces.

